# User Views on Online Sexual Health Symptom Checker Tool: Qualitative Research

**DOI:** 10.2196/54565

**Published:** 2024-11-04

**Authors:** Alicia Jean King, Jade Elissa Bilardi, Janet Mary Towns, Kate Maddaford, Christopher Kincaid Fairley, Eric P F Chow, Tiffany Renee Phillips

**Affiliations:** 1 School of Translational Medicine Faculty of Medicine, Nursing and Health Sciences Monash University Melbourne Australia; 2 Melbourne Sexual Health Centre Alfred Health Carlton Australia; 3 Department of General Practice The University of Melbourne Melbourne Australia; 4 Centre for Epidemiology and Biostatistics Melbourne School of Population and Global Health The University of Melbourne Melbourne Australia

**Keywords:** sexual health, sexually transmitted diseases, risk assessment, risk factors, smartphone apps, help-seeking behavior, health literacy, information seeking behavior

## Abstract

**Background:**

Delayed diagnosis and treatment of sexually transmitted infections (STIs) contributes to poorer health outcomes and onward transmission to sexual partners. Access to best-practice sexual health care may be limited by barriers such as cost, distance to care providers, sexual stigma, and trust in health care providers. Online assessments of risk offer a novel means of supporting access to evidence-based sexual health information, testing, and treatment by providing more individualized sexual health information based on user inputs.

**Objective:**

This developmental evaluation aims to find potential users’ views and experiences in relation to an online assessment of risk, called iSpySTI (Melbourne Sexual Health Center), including the likely impacts of use.

**Methods:**

Individuals presenting with urogenital symptoms to a specialist sexual health clinic were given the opportunity to trial a web-based, Bayesian-powered tool that provides a list of 2 to 4 potential causes of their symptoms based on inputs of known STI risk factors and symptoms. Those who tried the tool were invited to participate in a once-off, semistructured research interview. Descriptive, action, and emotion coding informed the comparative analysis of individual cases.

**Results:**

Findings from interviews with 14 people who had used the iSpySTI tool support the superiority of the online assessment of STI risk compared to existing sources of sexual health information (eg, internet search engines) in providing trusted and probabilistic information to users. Additionally, potential users reported benefits to their emotional well-being in the intervening period between noticing symptoms and being able to access care. Differences in current and imagined urgency of health care seeking and emotional impacts were found based on clinical diagnosis (eg, non-STI, curable and incurable but treatable STIs) and whether participants were born in Australia or elsewhere.

**Conclusions:**

Online assessments of risk provide users experiencing urogenital symptoms with more individualized and evidence-based health information that can improve their health care–seeking and provide reassurance in the period before they can access care.

## Introduction

Delayed identification and treatment of sexually transmitted infections (STIs) increases the likelihood of negative sequelae such as pelvic inflammatory disease, chronic pelvic pain, impaired fertility, pregnancy loss, infant morbidity and mortality, and neurological impairment [1,2]. In addition to individual outcomes, reducing the time interval between infection and identification is key to reducing transmission and community prevalence of STIs [3]. Variations in health care–seeking among people experiencing symptoms of STIs have been found, depending on the type of infection, level of engagement with, and distance to, specialist sexual health services [4].

Specialist sexual health services are under significant pressure to meet the rising demand for STI testing and treatment and remain difficult to access for many [5,6]. The local sexual health policy in Australia [5] and the World Health Organization’s *Global Health Sector Strategies on, Respectively, HIV, Viral Hepatitis and Sexually Transmitted Infections* [6] have emphasized the need for shared service delivery models and partnerships between specialist and primary health providers. General practitioners and pharmacists may be equipped to manage or advise in the treatment of common STIs (eg, chlamydia, gonorrhea, syphilis) and other genital infections (eg, candidiasis, tinea, balanitis, and bacterial vaginosis), and may be more geographically accessible than specialist sexual health services. However, some individuals experiencing urogenital symptoms lack confidence in raising sexual health questions with a general practitioner [7,8], including uncertainty about the general practitioner’s sexual health expertise [9].

Digital STI services offer a potential avenue for overcoming barriers to sexual health care, by facilitating access to evidence-based sexual health information, testing, and treatment [10]. Mobile apps providing STI information have proliferated but, concerningly, a review of 87 mobile apps providing STI information found that only 13 provided accurate information and 25 contained one or more pieces of potentially harmful information [11]. Online assessments of STI risk developed by sexual health researchers have been reported as helpful by users [12]. However, limited research has focused on their usability and impact on seeking health care [10]. Previous vignette studies from the field of cancer research suggest different risk communication formats influence health care decision-making but have not explored potential impacts on user experience [13,14].

The Supporting Timely and Appropriate Review and Treatment Online study sought users’ views on an online “symptom checker” tool, named iSpySTI (Melbourne Sexual Health Center [MSHC]) [15], that provides users with individualized information on common urogenital conditions and advice on health care–seeking. The objectives of the research were: (1) to explore user experiences of online assessments of STI risk and; (2) to understand the role of these tools in promoting timely and appropriate STI testing and treatment.

In this paper, “female” and “male” refer to people with vulvas and people with penises, respectively, while acknowledging that not all people with vulvas identify as female and not all people with penises identify as male.

## Methods

### Study Design

This developmental evaluation [16] used qualitative data collection and analysis methods to explore potential users’ views and experiences of using an online assessment of STI risk, particularly regarding the accessibility, acceptability, and usefulness of the tool. The study used a case study design [17,18], which involved comparing the actions and emotions described by individual participants (ie, cases) prior to and when using the tool. Comparison of shared and divergent experiences between cases informed the development of an explanatory model describing key factors influencing users’ experience of the symptom checker tool. Findings from the study will be used by the wider research team to modify future iterations of the tool. This study has been reported in accordance with the Consolidated Criteria for Reporting Qualitative Research (COREQ) guidelines [19] (Multimedia Appendix 1).

### Description of the Tool Development and Features

The symptom checker tool was developed by MSHC, a large, publicly funded sexual health clinic in the urban center of the capital city of the State of Victoria, Australia. The center provides free STI testing and treatment to all clinic attendees, with about 60,000 consultations per year.

The symptom checker is a web-based tool that generates a list of 2 to 4 potential sexual health conditions that might be causing a user’s symptoms, based on information they enter about themselves (eg, age, sex), their sexual practices, and their symptoms. The tool uses a predictive Bayesian model mathematical algorithm developed from a cross-sectional survey of 8318 participants, conducted at the MSHC during 2015-2016 [20]. Symptoms included in the tool were chosen based on a retrospective study of the most common presenting symptoms in 200 cases of each of 12 separate sexual health diagnoses. Additionally, common genital conditions not included in the clinic’s medical record system (ie, normal anatomical variants and tinea cruris) were included. Conditions included in the tool are shown in Table 1. Further details of the development of the tool are available in Towns’s [20] thesis.

**Table 1:** Conditions included in symptom checker tool by female and male genitalia.

Sex	Conditions included
Female	Pelvic inflammatory disease Urinary tract infection Genital warts Genital herpes Bacterial vaginosis Candidiasis Molluscum contagiosum Tinea cruris Normal anatomy variants
Male	Urethritis Urethral gonorrhea Genital warts Genital herpes Primary syphilis Balanitis Molluscum contagiosum Tinea cruris Normal anatomy variants

Users answer a series of multiple choice and numerical short answer (eg, age in years) questions including demographic information, risk factors for STIs (eg, number of sexual partners in previous 12 months and condomless sex), and their current symptoms. Questions about symptoms include the nature of symptoms (eg, pain, altered discharge, and lesions), their location, and appearance. Subsequent symptom questions and possible responses are individualized by previous answers. For example, females are shown line drawings of female anatomy, and only users reporting lesions are shown photos of lesions. Examples of questions are shown in Multimedia Appendix 2.

Results are reported as the percentage of similar MSHC attendees reporting similar symptoms that were diagnosed with 2 to 4 conditions. The accuracy of the tool’s predictions varies across conditions and sex, with sensitivity of 80% for bacterial vaginosis and candidiasis in females and 95% in other conditions, and specificity between 38% (primary syphilis in men) and 76% (urinary tract infections in males and females) [20]. All users are encouraged to seek clinical confirmation of results. The results page also includes hyperlinks to online “fact sheets,” advice on testing, treatment, and partner notification, and a “referral letter” that can be taken to a general practitioner (Multimedia Appendix 3). User inputs and the results page are not stored by the site.

### Sampling

A convenience sample of individuals (aged greater than 18 years) presenting to MSHC with urogenital symptoms was recruited via MSHC clinicians. As recruitment progressed, invitations were purposively directed to certain demographic groups (eg, heterosexual men) and presentations (eg, genital lesions) to ensure a diverse sample of participants.

### Recruitment and Consent

Potential participants were given a flyer with a quick response code web page link to the symptom checker tool and advised they could try the site with or without participating in the research. At the end of the tool, users were invited to provide their contact details should they wish to participate in a semistructured interview to provide more detailed feedback. The use of the tool did not alter the delay or change the care individuals received at MSHC, and their results were not provided to their treating clinicians unless spontaneously mentioned by the participant in subsequent clinical encounters.

Respondents were contacted by AJK via email or telephone and provided with verbal and written information about the study. A mutually convenient time was arranged for an interview during which informed consent was obtained. Of the 18 MSHC attendees who expressed an interest, 15 were able to be contacted, and 1 withdrew before consent as they did not feel comfortable discussing their sexual practices in a research interview.

### Data Collection

Once-off interviews were conducted from June 2023 to August 2023 by AJK in person at MSHC, over the phone, or via Zoom (Zoom Video Communications) videoconferencing. No others were present during the interviews conducted at MSHC and prior to Zoom and telephone interviews, participants were advised of the sensitive nature of the interviews and it was strongly suggested they access a private space.

AJK is a cisgender female research fellow with a PhD and experience in qualitative research with health service users. Prior to the interview, she had no relationship with participants. AJK explained to the participants she is not a sexual health clinician and that her interest in the research is improving experiences of sexual health care seeking.

#### Demographic Questionnaire

Before the interview, a demographic questionnaire was administered to participants by AJK (Multimedia Appendix 4).

#### Semistructured Interviews

An interview guide was used (Multimedia Appendix 5) covering participants' views on, and experiences of using the symptom checker tool. Participants were also asked if they would use the site again, how it might influence their health care–seeking behavior, and how it might be promoted to others. Interviews were audio recorded and transcribed by a professional transcription service based in Australia. No participants requested an interview transcript but 12 accepted the offer of an interview summary after initial coding with no changes suggested by the 3 participants that responded. Field notes were recorded by AJK following each interview.

### Data Analysis

Consistent with a case study design, data analysis used a bespoke combination of established qualitative data analysis methods relevant to the study question and described by authors in the field [21-23]. Data analysis occurred concurrently with data collection to allow for the exploration of findings from earlier interviews with subsequent participants. Interview transcripts were checked for accuracy and then manually coded 3 times by AJK using descriptive, action, and emotion coding [21]. NVivo 14 software (QSR International) was used to manage the data, including transcripts, field notes, interview summaries, and analytic memos and to retrieve data allocated to codes and individual cases.

Action and emotion coding was used to inductively identify the semantic and latent meaning in participants’ responses. Action (or process) coding [21] involved allocating gerunds (ie, -ing words) to sections of transcript texts pertaining to participants’ process from noticing potential symptoms of an STI to seeking health care, including their imagined process if using the symptom checker tool (eg, seeking information online, jumping to conclusions, waiting it out, coming into the clinic). Emotion coding [21] complemented this analysis by identifying the emotions experienced by participants throughout the process. This combination of coding methods was chosen in recognition of the relationship between cognitive processes and actions (as captured by action or process coding), emotions, and health care–seeking. Analytic memos [22] were written summarizing action and emotion coding for each participant.

Key concepts identified during coding were mapped into a graphical display [23] which was progressively refined after 6, 10, and 14 interviews to the flow diagram shown in the results below. Discussions with the research team at each of these time points supported the refinement of themes, reflexivity, and recruitment of a diverse sample. Following 14 interviews, the team agreed sufficient interviews had been conducted to provide a meaningful explanation of the tool’s usefulness and potential impacts. Action codes relating to each of the key concepts were grouped into coding sets. Data within these sets were then reread and summarized into concept descriptions [22] which are included in the results. An example concept description is provided in Multimedia Appendix 6.

Emotion coding alerted researchers to differences in the emotional impact of using the site reported by participants. Individual case comparisons [23] were used to identify factors predicting differences in the reported emotional impact of the site. This involved creating a matrix display of case attributes (eg, sex, sexual identity, site results, clinical diagnosis) and looking for common factors that predicted a certain emotional outcome.

### Ethical Considerations

Ethical approval was obtained from Alfred Hospital Ethics Committee, Melbourne, Victoria, Australia (approval 39-23) and Monash University (project 37313). All data were deidentified and stored on a secure server accessible only to the researchers. Participants received financial compensation of Aus $50 (approximately US $33 at the time of the study) as an e-gift voucher for their participation.

## Results

### Participant Demographics

Interviews of 29-50 (mean 39, SD 6) minutes were conducted with 14 sexual health service users of diverse ages, genders, sexual identities, and countries of birth. Participants’ demographic information as shared in their responses to the demographic questionnaire is reported in Table 2.

**Table 2:** Participant demographics (N=14).

Category			Participants, n (%)
**Age (years)**			
	18-29	10 (71)	
	30-39	3 (21)	
	40 or older	1 (7)	
**Gender**			
	Woman	8 (57)	
	Man	6 (43)	
	Nonbinary	0 (0)	
**Recorded sex at birth**			
	Female	8 (57)	
	Male	6 (43)	
**Sexual identity**			
	Bisexual	6 (43)	
	Straight (heterosexual)	5 (36)	
	Gay	2 (14)	
	Queer	1 (7)	
**Region of birth** ^a^			
	Australia	3 (21)	
	Mainland South East Asia	1 (7)	
	Maritime South East Asia	1 (7)	
	New Zealand	2 (14)	
	South America	1 (7)	
	Southern Asia	3 (21)	
	Southern Europe	1 (7)	
	United Kingdom	2 (14)	
**Sex work in the past 12 months**			
	Yes	2 (14)	

^a^As defined by the Australian Bureau of Statistics.

### Interview Findings: Influence on Pathway to Sexual Health Care

**Figure 1:**
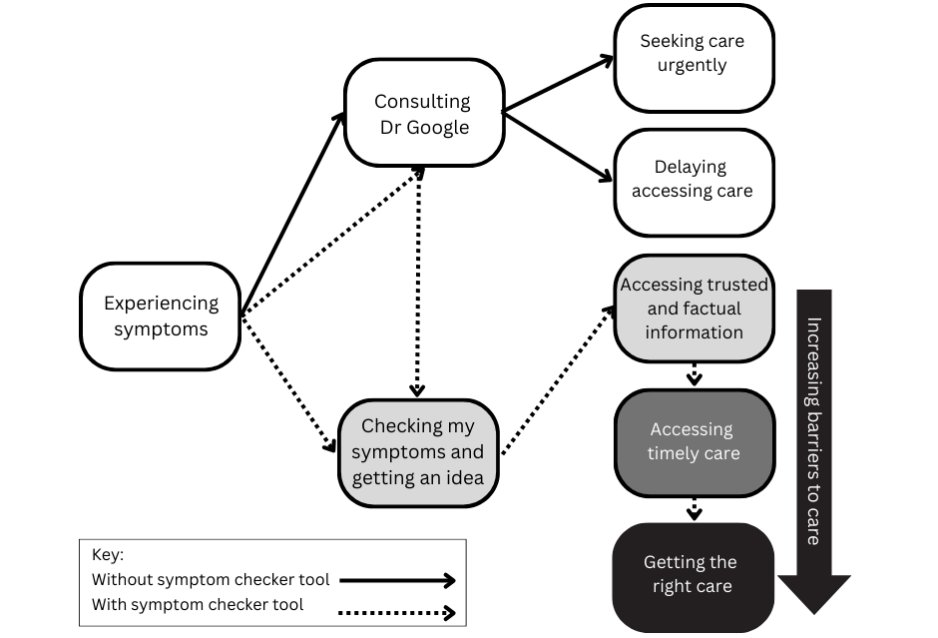
Pathways to care suggested by interview findings.

Participants described their current pathway to sexual health care and how they imagined this would change if they had access to the symptom checker tool. These pathways are shown in Figure 1. Steps in the pathway to care are described in more detail in the text below with exemplar quotes. Participant names are pseudonyms.

#### Experiencing Symptoms

Participants described experiencing urogenital symptoms as sensory, cognitive, and emotional experiences. They were alerted to something different in their bodies by sensations such as smell, sight, and pain. Not knowing the cause of the change created a cognitive load resulting in people being preoccupied. The uncertainty also created a heightened emotional state which motivated them to seek out information online or from health care professionals.

It’s so easy to have a bit of an itch down there and then I’d be “Oh my God, I’ve got HIV,” or something. Your mind always jumps to those sorts of things.

Costa, bisexual woman

#### Consulting “Dr Google”

Participants sought information online when they were concerned about urogenital symptoms before accessing care. Participants reported, to varying degrees, consulting internet search engines to “do their research” prior to accessing clinical care. The search results included both relevant and irrelevant information causing confusion and overwhelming doubts, despite many participants describing seeking out more reputable sources. The inclusion of irrelevant conditions and worst-case outcomes resulted in escalating anxiety.

Because the first thing people are going to do is Dr. Google and everything's going to come up as cancer or you’re dead. So, that anxiety level is already going to be high for someone that hasn't experienced this before.

Kiba, gay man

#### Seeking Care Urgently

In the context of the many possible conditions identified by a general internet search of symptoms, many participants described a sense of urgency to access clinical care to rule out the worst-case scenario.

…because sometimes you cannot get immediate access to a clinic or you just want to know what you could possibly have first before jumping to conclusions or scaring yourself for nothing.

Rondell, straight woman

#### Delaying Accessing Care

Some participants misattributed their symptoms to non-STI causes, postponing care in the belief symptoms would resolve without treatment.

I think the reason people take their time to come to the clinic is because they think, “This is something that will fix itself on its own.” But if they know from somewhere that, “No, this thing needs treatment.” . . . For the syphilis thing, it took me eight or nine days to come here because I was under the impression that this was an injury.

Mayu, straight man

While, in some instances, participants postponed seeking treatment thinking symptoms would resolve themselves, Mayu had also postponed it for fear he had an incurable STI.

Mayu: I think if I have something and I know that, “Okay. I’m going to die” . . . Usually it’s this thing in the mind. . . “This is not curable at all and this will last forever and it will stay like this.” So, your mind is like, “Is there a treatment or not? If I’m not seeing anything, I’m not doing any extra research or something like that, then I would be just like, “No.”

AJK: You’d just ignore it?

Mayu: Yes.

#### Checking My Symptoms and Getting an Idea

The symptom checker tool provided a more attractive alternative to internet search engines in that it provided a narrow list of potential causes of urogenital symptoms and facilitated access to more measured information. All participants who used the tool said that they would use it again in the future.

Often, you can Google your symptoms and [it] just send[s] you down a spiral of X, Y, and Z of what you could have and maybe this is a bit more level-headed advice or consultation on the possibilities.

Jay, bisexual man

Users were circumspect in terms of their expectation of the site’s accuracy, describing it as “getting an idea” rather than providing a definitive diagnosis.

I don’t, obviously, expect the [symptom checker tool] to know because I know that it’s going to be like a general kind of answer. So, in a way, I’m kind of satisfied with that because it’s narrowed it down from however many there actually are to what I can see on the screen now.

Giani, straight man

Though they preferred the symptom checker tool to internet search, they recommended search engine optimization as a key strategy for promoting the site, as a search engine was their first port of call when symptoms arose and the most likely way of finding and accessing before accessing the site.

As shown in Figure 1, outcomes of using the symptom checker tool included accessing information that had the potential to facilitate access to timely care and the right care. The latter 2 outcomes were not expected by all participants due to other barriers to care such as health care costs, distance from specialist services, and confidence in self-advocacy.

#### Accessing Trusted and Factual Information

The tool was seen as a trustworthy source of information as it was linked to the MSHC clinic.

I always look for stuff attached to a clinic or a university or some sort of institution which has a bit of backing to it. It gives me a little bit of comfort, knowing that it’s coming from something like that.

Leigh, bisexual woman

Moreover, the information provided by the tool was based on users’ inputs.

The pic[ture]s are like something that I have and the questions that were there, like fluid coming out [of my penis], so I know this is also my condition. So, the output I’m seeing is based on the input I give, so that makes me trust it.

Mayu, straight man

Accessing more individualized, trusted, and factual information was reassuring in the intervening period between experiencing symptoms and accessing care.

I think having the information just stated plainly with just facts and stats and resources it can dull the anxiety a little bit. Because I guess it’s factual, you are like okay, this is fine, I’m not going to... a few days isn’t going to... it’s not the end of the world.

Edi, bisexual woman

Information provided by the tool had the potential to facilitate access to other sources of information, directly through hyperlinked information and indirectly by users searching for more specific information online. Participants described that greater awareness of urogenital symptoms and their causes might improve future help-seeking and support peer education. Those perceiving the discussion of sexual health in their country of origin as a social taboo, such as Adam, noted the site provided access to information they had not previously encountered, overcoming a potential barrier to care.

I think people growing up in Australia, they are already aware about their sexual health, and they are more comfortable talking about it than people who are from the other countries. Because, in other countries, it’s not an issue that people usually talk about. . .. so, they don’t get the information. So, I think this site could give them information.

Adam, gay man

#### Accessing Timely Care

While participants found the site useful and trustworthy, they would still seek medical care after using the site.

It feels like somewhere in between a Google search and talking with a professional. . .. I mean, there’s always going to be a little bit of suspicion. I wouldn’t trust it 100 per cent. I’d probably just want to see some test results.

Jay, bisexual man

Information provided by the site informed the urgency with which users would seek care. If an STI was identified, users reported they would be motivated to seek care sooner. Being able to access this information, without attending a clinic, meant users would access care when symptoms were mild rather than waiting for them to worsen and encountering barriers, such as high demand and restricted opening hours at free clinics.

I was very scared because I had strong symptoms. My uterus was in pain and I was bleeding. So, they asked me to wait but they couldn’t help me that day because it was too busy and it was Friday. So, I had to wait until Monday . . . At the beginning, I started to feel symptoms but . . . it was a very small pain, so I wasn’t concerned. . .. If I had access to the website, I would go before the pain gets so bad.

Donovan, bisexual woman

Participants reported they would still seek care for possible non-STI causes of genital symptoms (eg, bacterial vaginosis) but would do so with less urgency, avoiding disruption to work or study plans.

Seeing that percentage that there was just BV and thrush I would probably go anyway, but maybe not immediately, maybe the following day, because I was in a little bit of a panic. I thought, “Oh my God, what do I have?” I would be like, “Ah, okay, maybe it’s just BV. I can go Monday.” Without changing all my plans for tomorrow.

Jane, bisexual woman

#### Getting the Right Care

To varying degrees, participants described the potential use of the site in supporting them to access better sexual health care. Participants born in Australia who preferred to seek care from specialist sexual health services described feeling comfortable using the results to support them in seeking appropriate sexual health care from general practitioners.

My first preference would be to go to the [sexual health] clinic but I just don't have the time to either travel there or to wait in the queue to be seen. . .. If I can get to the clinic, I will go straight there and explain my symptoms knowing that these people deal with this day in day out. If I don't have the time to go to the clinic then I would use this and take it to a GP.

Kiba, gay man

Participants born in Australia described potentially using the information provided by the site to support improved communication with health care professionals.

I think sometimes when I talk to like a GP I forget to mention things in person. Whereas if you’re doing a questionnaire it prompts you to record all your symptoms and asks all the right questions. I think it definitely provides that reassurance, bringing this results letter to the doctor or to the GP, because then you know you won’t have missed anything.

Edi, bisexual woman

In addition to communicating their symptoms clearly, participants born in Australia reported the site results might support self-advocacy.

As a woman, so many male doctors don’t listen to you. To have an official page that’s from other experts, I think, is so good to back you up. I’ve definitely gone to the doctors with symptoms of STIs and they’ve been – “Oh, it’s just your period. It’s just being a woman.” Male doctors can discredit women a lot, so I think it is very helpful. . . to back you up and support what you’re saying, that actually, no, it’s not just me being a woman, I have this. The website says so.

Costa, bisexual woman

Participants from countries other than Australia were less likely to be comfortable using the tool to support accessing care from a general practitioner. Partly this was due to eligibility and cost barriers but those able to attend general practitioners expressed discomfort with self-advocacy, particularly with unfamiliar health care providers.

If I was doing it at my current GP that would be fine because I know her. . .. But perhaps if it was a doctor I didn’t know I wouldn’t feel comfortable doing that.

Binh, bisexual woman

In addition to improving access to sexual health information and quality care, participants reported potential impacts on their emotional well-being when using the symptom checker tool.

### Interview Findings: Emotional Impact of Use

Comparative case analysis of the explicit and implicit emotional content of participants’ testimonies revealed differences related to the possible causes of urogenital symptoms identified by the site. The results of this analysis are summarized in Table 3. Briefly, participants imagined that (had they accessed the tool prior to attending the clinic) it would have offered reassurance in the intervening period between noticing symptoms and seeking care, in the circumstance that it identified a likelihood that their symptoms were caused by a non-STI (eg, candidiasis, urinary tract infection) or that they had a curable STI (ie, bacterial infection). The exceptions to this outcome were when a new potential incurable but treatable STI (eg, herpes) was identified or the tool was unable to provide a result. In both circumstances, participants would have remained concerned about their symptoms and would have sought care to confirm the result.

**Table 3:** Case comparison of possible conditions identified by site, clinical diagnosis, and emotional impact.

Participant pseudonym		Possible conditions identified by site	Clinical diagnosis	Imagined emotional outcome, if using site prior to care	Outcome	
**Case group summary: non-STI cause of urogenital symptoms identified by site and confirmed clinically**						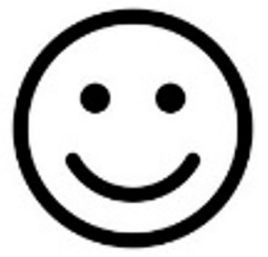
	Rondel	non-STI^a,b^	non-STI	Reassured		
	Jane	non-STI	non-STI	Reassured		
	Costa	non-STI	non-STI	Reassured		
	Leigh	non-STI	non-STI	Reassured		
	Binh	non-STI	non-STI	Reassured		
**Case group summary: curable STI identified as possible cause of urogenital symptoms by site and confirmed clinically**						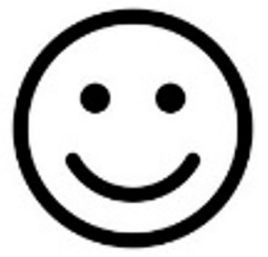
	Mayu	Curable^c^ STI	Curable STI	Reassured		
	Jay	Curable STI	Curable STI	Reassured		
	Donovan	Curable STI	Curable STI	Reassured		
	Edi	Curable STI	Curable STI	Reassured		
	Giani	Curable STI	Curable STI	Reassured		
**Case group summary: recurrence of incurable but treatable STI identified as possible cause of genital symptoms by site, confirmed clinically**						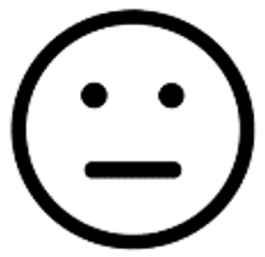
	Kiba	Incurable^d^ STI	Recurrence of incurable STI	Not concerned		
**Case group summary: incurable but treatable STI identified as possible causes of genital symptoms and confirmed or disconfirmed clinically**						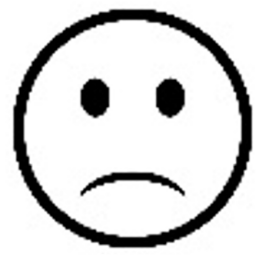
	Sunny	Incurable STI	non-STI	Not reassured		
	Felix	Incurable STI	Incurable STI	Not reassured		
**Case group summary: no condition identified as symptoms not able to be entered. Non-STI diagnosed on clinical examination**						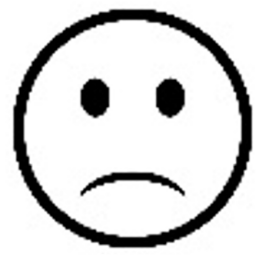
	Adam	No result	non-STI	Not reassured		

^a^STI: sexually transmitted infection.

^b^Genito-urinary conditions not caused by a sexually transmitted infection (eg, candidiasis, balanitis).

^c^Able to be cured with antibiotics (eg, syphilis, chlamydia, *Mycoplasma genitalium*, gonorrhea).

^d^Incurable but treatable (eg, herpes simplex virus, human papillomavirus).

As described earlier by Jane, the identification of non-STI causes of urogenital symptoms (eg, bacterial vaginosis, candidiasis, and urinary tract infections) was experienced as reassuring. Somewhat surprisingly, however, the identification of curable STIs (eg, chlamydia, syphilis) was similarly reassuring.

The first time I came [to MSHC], I didn’t know anything about [STIs] – at that time, if I had had this app and . . . it showed that syphilis and a picture of it and then I say, “Oh, this is the one I have right now” and next to it, if it’s treatable or not, so then I can be like, “Okay. This thing is treatable” and that would be a big help and a relief for me.

Mayu, straight man

Identification of a possible incurable but treatable STI (eg, herpes) was concerning for users, in the context of misinformation surrounding herpes, but not taken as a definitive diagnosis.

It's kind of scaring me a bit. . .. when I pick a [response] that I have a lump on my vagina it’s showing something STI related. . .. So, I actually have this cyst, which is not a venereal disease. . . [The symptom checker tool] said “8% women who have your symptoms are diagnosed with herpes.” I mean, I was shocked, not much, to be honest, because I know it’s computer generated. I see where it led [to that result], as well.”

Sunny, straight woman

Moreover, the percentage provided to users, of MSHC attendees with similar sexual practices and symptoms diagnosed with a condition, provided hope in the intervening period between noticing symptoms and clinical diagnosis.

I think it's having that statistic because when you get worried about it you think, “Oh crap, that's it, that's almost certainly X,” and to realize that - this is possibly this. . . but there's still a good chance that it's benign or less of an issue.

Felix, straight man

Participants facing a potential herpes diagnosis described a need for practical information about potential implications for their future sexual life.

I think some sort of full and frank, “Hey look, [a herpes diagnosis is] not the end of your sex life, it's just that it’s going to have to change and you need to be thinking about protecting other people and having that upfront conversation about it” . . . because that to me is thinking, “Well crap, is that it? I either have to lie or my sex life is over?”

Felix, straight man

Users whose symptoms did not appear on the site and received only general information on asymptomatic STI testing would not have been reassured without clinical confirmation.

So, the symptoms that they showed, I haven’t experienced any of those symptoms. So, the site told me that “You don’t have any…” Like. . . there was no diagnosis. . .. But because I already did the test at MSHC, I was okay.

Adam, gay man

## Discussion

### Principal Results

This developmental evaluation of an online sexual health “symptom checker” tool was conducted to gain insight into its potential influence on experiences of health care–seeking. The findings suggest the tool has the potential to support improved access to accurate sexual health information and timely and best practice sexual health care. Moreover, access to more specific information was generally beneficial to well-being in the intervening period between experiencing symptoms and accessing care.

Sexual health service users who used the tool reported its superiority to current avenues of seeking online sexual health information, namely internet search engines. Participants saw the tool as a source of more trustworthy and measured information. The specificity and probabilistic reporting of possible causes of urogenital symptoms were found to be more reassuring compared with a general internet search, which could find numerous and mostly irrelevant possibilities. The prominent use of search engines as the first step in people’s pathway to seeking health care, suggests search engine optimization might be a worthwhile focus for the promotion of tools such as the symptom checker. As well as being suggested by existing sexual health service users, making the tool easier to find on Google may also direct people who would otherwise not have sought health care, due to overwhelming information or lack of service information, into care.

While the symptom checker tool results page invariably advised seeking clinical confirmation of results, participants described a variable influence on the urgency of health care seeking. Those who may have previously urgently sought care to exclude an STI or bloodborne virus (eg, HIV) were reassured they could attend at their earliest convenience, whereas those with potential STI symptoms were encouraged to seek care sooner. These findings suggest the tool may have a role in avoiding the negative sequelae of delayed STI treatment for individuals (eg, impairment of reproductive function, infant morbidity and mortality, neurological impairment) [1,2] and the wider community [3]. Modification of digital health tools to provide more tailored sexual health advice is not feasible within the current regulatory environment in Australia. Further, community-based implementation and outcome-focused research are needed to gain the confidence of the public and regulatory bodies before these tools can be more usefully integrated into existing health care systems. Moreover, it is not the tool’s purpose to direct people away from sexual health services but to encourage earlier presentation, thus reducing transmission and downstream pressures on overburdened sexual health services.

Australian-born participants reported they would use the symptom checker tool before seeking sexual health care from general practitioners. Barriers to seeking sexual health care from general practitioners over specialist sexual health clinics include copayment costs but also lack of confidence on the part of both people seeking care and providers [7,24]. As such, this tool may support individuals who have the financial means to feel confident in accessing sexual health care from a general practitioner rather than a specialist sexual health service, reducing pressure on sexual health clinics and overcoming the tyranny of distance for individuals in rural and remote locations [5,6]. While differences in social norms between Australia and their country of origin were noted by some participants as limiting their prior access to sexual health information, these should not be seen as representative of the diversity of experience in either context. Moreover, comfort in self-advocacy with health care providers reported by Australian-born participants could be reflective of the sample rather than Australian-born people, in general.

An unexpected finding of the research was the potential positive impact on users’ well-being. Experiencing urogenital symptoms was an anxiety-provoking experience for many, especially young people. Participants described being able to access trusted and factual information about possible causes of urogenital symptoms in the intervening period between noticing symptoms and being able to access care, which was reassuring, even when an STI was identified. The exception to this was when a potentially incurable but treatable STI (eg, herpes) was identified. While not seen as a definitive diagnosis, the provision of practical information was key to processing these common but stigmatized diagnoses [25]. These findings, and the direct suggestions of study participants, underscore the need for educational campaigns to reduce misinformation about herpes.

### Limitations

This study sought the views of attendees to a public sexual health clinic in an urban setting on a tool developed for this population. While efforts were made to recruit a diverse sample, we were unable to recruit any transgender or gender-diverse people, women who have sex with women only, or First Nations people, groups who are not strongly represented in MSHC attendees. Moreover, as suggested by findings in relation to accessing timely care and getting the right care, users not engaged with care may face additional barriers that alter the experience and outcomes of using symptom checker sites. Findings should, thus, be interpreted with caution when considering their application to other potential users. Planned modifications and user testing of the updated symptom checker tool will aim to improve its accessibility to transgender and gender-diverse users. Sampling bias associated with participant self-selection is likely to have occurred.

### Comparison With Prior Work

This research adds to our understanding of the usefulness of a growing suite of digital STI and HIV services such as self-testing and self-sampling, treatment and referral, partner notification, and prevention [10]. Existing research promoting engagement of target populations with sexual health services via digital interventions has largely focused on the promotion of asymptomatic testing via online self-testing [26,27] rather than the provision of individualized information about risk. In a previous survey conducted by the authors on the Check Your Risk site, which provided a risk assessment of common bacterial STIs (eg, chlamydia, gonorrhea, syphilis) and bloodborne viruses (HIV, hepatitis B, and hepatitis C) based on risk factors only (ie, no symptoms), 70% of users reported the site was useful [12]. However, no further information on user experience was sought. This study is the first to report findings of a tool providing information about STI risk to health service users experiencing STI symptoms. It also contributes insights into the user experience of these tools, a gap identified by a review by Tucker et al [10]. Future development of the “symptom checker” tool will incorporate feedback, provided by the participants, to improve the appearance, accessibility, usability, and information provided by the current user interface.

### Implications for Practice

The findings of this research suggest that symptom checker tools such as iSpySTI may hold value in directing people to the right care at the right time. Examples of the integration of digital sexual health tools with in-person services are emerging in the literature [28,29], adding to the growing body of literature on pilot e-STI services [30-32]. Service commissioners and research funders can bolster the confidence of regulators by funding implementation-focused research into the successful integration of digital and in-person sexual health services.

### Conclusions

The improved control of STIs requires novel strategies for improving equity in access to sexual health information and services. The unprecedented availability of sexual health information to people experiencing urogenital symptoms via the internet has potential disadvantages such as information overwhelm, misinformation, and escalation of distress before health care can be accessed. Online assessments of risk provide users with more individualized and reliable information, which may in the future support access to care across digital, primary care, and specialist sexual health settings.

## Appendix

Multimedia Appendix 1 Completed COREQ (COnsolidated criteria for REporting Qualitative research) Checklist.

Multimedia Appendix 2 Example initial question shown to male users.

Multimedia Appendix 3 iSpySTI referral letter.

Multimedia Appendix 4 Demographic questionnaire.

Multimedia Appendix 5 Semistructured interview guide.

Multimedia Appendix 6 Example concept description.

## Abbreviations

COREQ: Consolidated Criteria for Reporting Qualitative Research
MSHC: Melbourne Sexual Health Center
STI: sexually transmitted infection
