# Supplementary material for: User Views on Online Sexual Health Symptom Checker Tool: Qualitative Research
Source: JMIR Form Res. 2024 Nov 4;8:e54565. doi: 10.2196/54565 (PMC11574491; doi:10.2196/54565)
Supplement: Multimedia Appendix 2 [file formative_v8i1e54565_app2.doc]

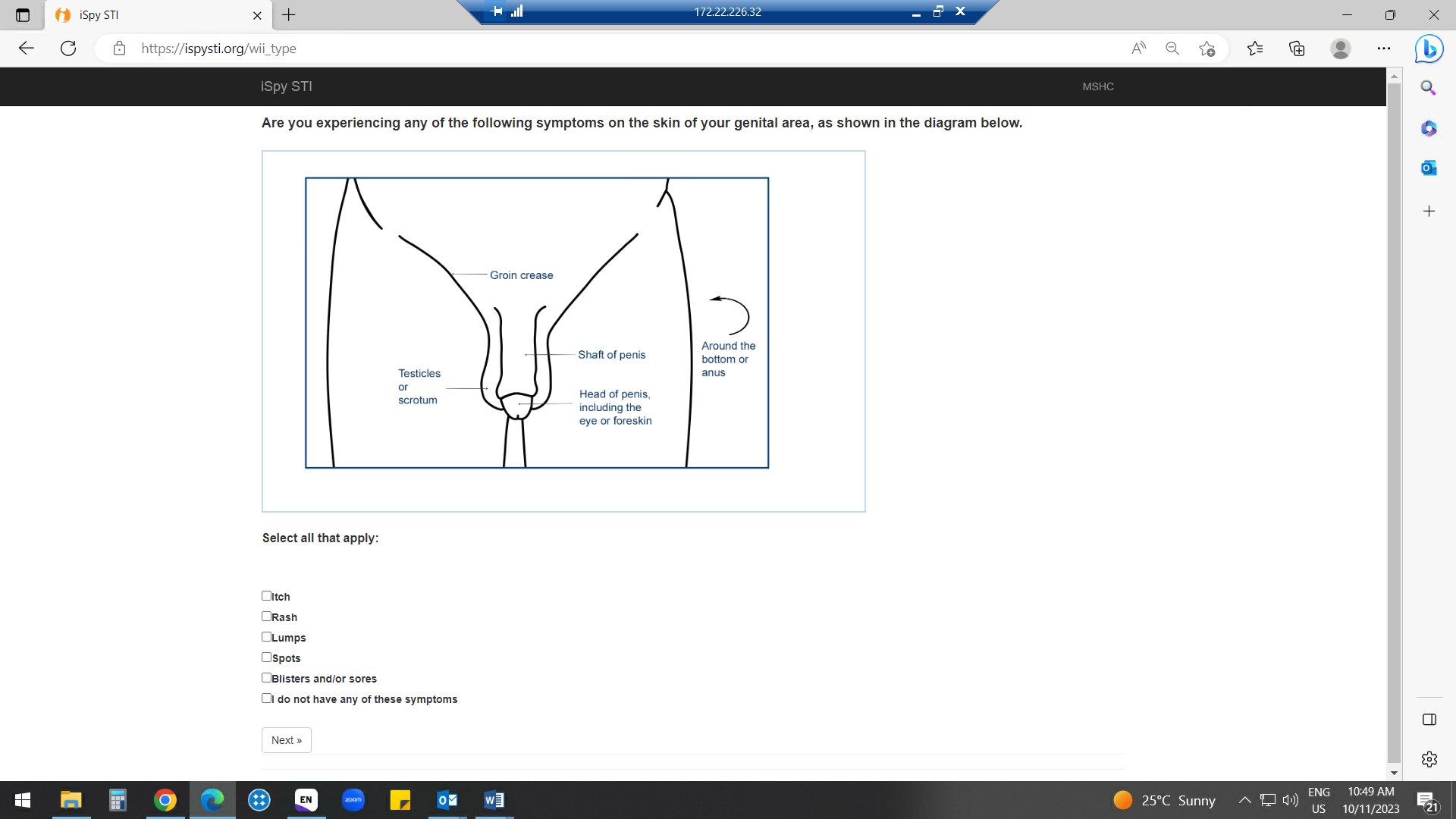


Figure 1. Example initial question shown to male users


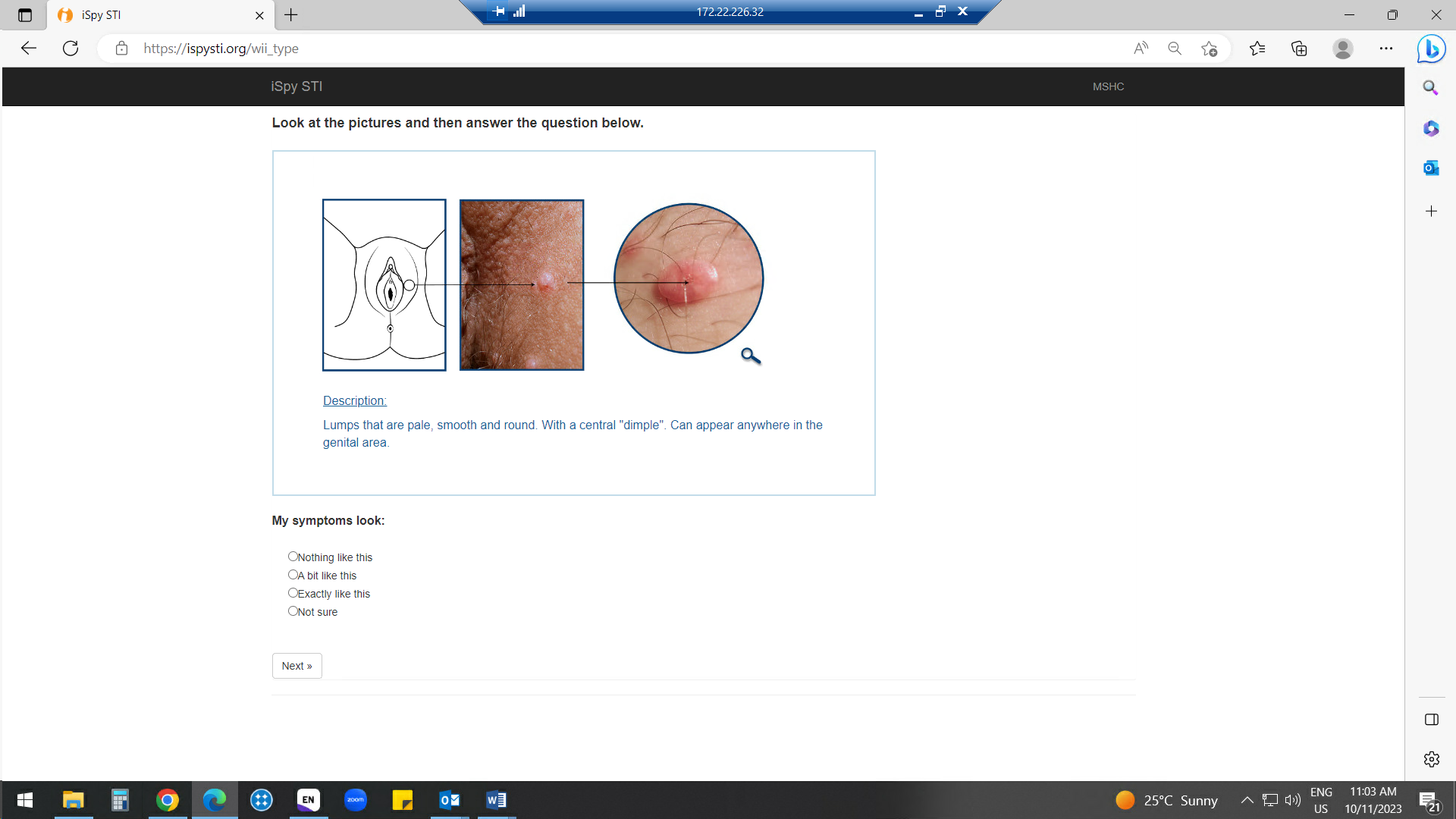


Figure 2. Example question shown to female users reporting "lumps"
