# Supplementary material for: User Views on Online Sexual Health Symptom Checker Tool: Qualitative Research
Source: JMIR Form Res. 2024 Nov 4;8:e54565. doi: 10.2196/54565 (PMC11574491; doi:10.2196/54565)
Supplement: Multimedia Appendix 3 [file formative_v8i1e54565_app3.pdf]

580 Swanston Street, Carlton 3053
Phone: (03) 9341 6200; 1800 032 017
Fax: (03) 9341 6279 TTY: (03) 9347 8619
www.mshc.org.au

12 March 2024

STI Risk Report

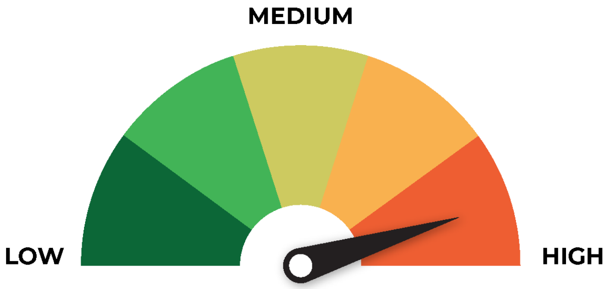

High risk

Based on your responses, you have been identified as **high risk** of STIs.
We recommend that you **get tested** as soon as possible.

We have generated the risk of the four infections for a person with the same answers that you gave but who attended Melbourne Sexual Health Centre. These risks are below.

Risk of HIV

- **Their risk of HIV is high**
- At present, in a group of **1000 people**, **4** will have HIV.
- Over the next 12 months, in a group of **1000 people**, **less than 1** are likely to catch HIV.

Risk of Syphilis

- **Their risk of syphilis is high**
- At present, in a group of **100 people**, **11** will have syphilis.
- Over the next 12 months, in a group of **100 people**, **4** are likely to catch syphilis.

Risk of Gonorrhoea

- **Their risk of gonorrhoea is high**
- At present, in a group of **100 people**, **27** will have gonorrhoea.
- Over the next 12 months, in a group of **100 people**, **12** are likely to catch gonorrhoea.

Risk of Chlamydia

- **Their risk of chlamydia is high**
- At present, in a group of **100 people**, **15** will have chlamydia.
- Over the next 12 months, in a group of **100 people**, **10** are likely to catch chlamydia.

Reduce my risk recommendations

You can reduce your risk or harm from these infections by:

- Having regular tests to detect infections early (reduce complications)
- Using condoms for vaginal or anal sex (reduces HIV or STI risk 90-95%)
- Taking PrEP if you are at high risk of acquiring HIV (see https://www.pan.org.au/)(reduce HIV risk by 99%)

In addition, a person can reduce the harm from these infections by testing

Benefits of testing

- Prevents complications from the infections by early treatment
- Prevents unknowing transmitting the infection to others

Without timely treatment, you may develop complications from the infections including

- Infertility (untreated chlamydia or gonorrhoea)
- Chronic pain (untreated chlamydia or gonorrhoea)
- Loss of hearing or sight (untreated syphilis)
- Cancer (untreated HIV)
- Higher risk of complications from untreated HIV infection such as cancer or infections (treatment of HIV is safe and easy and prevents these complications)

Where to go

If you consider yourself at risk, we recommend you take your results to your General Practitioner. To find General Practitioners click here.

It is essential that you see a doctor if you have any genital, anal, or physical symptoms, or if you think you could have an infection. If one of your sexual partners has told you that they have an infection, let your doctor know as it may assist with your care.

These recommendations are only intended to be a guide and may not necessarily suit your individual circumstances. If you have been tested for STIs recently, you may not need to be retested again.

Further information

For your interest and information, sexual health fact sheets can be found here. Treatment guidelines for sexually transmitted infections can be found here. Partner notification information can be found here.

This report has been generated by the MySTIRisk web application on the Melbourne Sexual Health Centre website. We applied machine-learning approach using retrospective data from thousands of clients attending our service, to generate the most likely and other possible diagnoses. These suggested diagnoses do not substitute professional medical advice or consultations with healthcare professionals.

ID: 8a1797ed-c99a-454f-9a36-ddbec1bc6e0f
