# Supplementary material for: User Views on Online Sexual Health Symptom Checker Tool: Qualitative Research
Source: JMIR Form Res. 2024 Nov 4;8:e54565. doi: 10.2196/54565 (PMC11574491; doi:10.2196/54565)
Supplement: Multimedia Appendix 4 [file formative_v8i1e54565_app4.docx]

**Demographic questions**

*The following questions are for the purposes of describing general participant characteristics. Information provided will be used to describe the participants and the groups to which they belong but care will be taken in reporting not to identify individual participants.*

1. How old are you? ______________________________
2. How do you describe your gender?

- Woman or female
- Man or male
- Non-binary
- I use a different term
  - Please specify ______________________________________
- Prefer not to answer

1. At birth, you were recorded as:
   - Male
   - Female
   - Another term
     - please specify______________________________________
   - Prefer not to answer (inclusion optional)
2. How do you describe your sexual identity?

- Straight (heterosexual)
- Gay or lesbian
- Bisexual
- I use a different term
  - Please specify______________________________________
- Don’t know
- Prefer not to answer

1. Were you born in Australia?

- Yes
- No
  - Which country were you born in? _______________________
  - How many years have you lived in Australia? ______________
- Prefer not to say

1. Are you Aboriginal or Torres Strait Islander?
   - Aboriginal
   - Torres Strait Islander
   - Aboriginal and Torres Strait Islander
   - Prefer not to say
2. Have you engaged in sex work in the past 12 months? Yes / No
3. Are you living with HIV? Yes / No
4. Would you like to select your own pseudonym, which will be used to distinguish your responses from other participants and protect your anonymity?
   - Yes, please refer to me as:______________________
   - No, I agree to the researcher assigning me a name.
