# Supplementary material for: User Views on Online Sexual Health Symptom Checker Tool: Qualitative Research
Source: JMIR Form Res. 2024 Nov 4;8:e54565. doi: 10.2196/54565 (PMC11574491; doi:10.2196/54565)
Supplement: Multimedia Appendix 5 [file formative_v8i1e54565_app5.docx]

**Semi-structured Interview questions**

*NB. These questions are intended to provide a rough guide of topics to be covered and do not preclude the interviewer from pursuing lines of enquiry that emerge from participant responses that may contribute to the overall research question and aims.*

1. What did you think of the MySTIRisk website? *Prompt, if necessary:*
2. What’s your understanding of the purpose of the website?
3. What did you think of the look and feel of the website?
4. How would you describe your overall experience using and navigating the website? *Prompt if necessary:* How did you find answering the questions?
5. How did you feel after reading your risk report? *Prompt if necessary:*
   1. How easy was it to understand?
   2. How useful was the information in the report?
   3. How relevant was it to you?
   4. Do you trust the information and advice provided in the risk report? Why?
6. What action, if any, have you taken or will you take after reading your risk report? Why?
7. When the MySTIRisk website becomes available on the MSHC website, would you use it?

If yes:

- 1. How and when would you use it?
  2. If you could change anything on the website, what would it be and why?

If no:

1. Why not?
2. Are there any changes you can suggest that would make you more likely to use it?
3. If the changes you have suggested can be made, is there anything else that would stop you from using the site (e.g., cost, privacy concerns, if it were an app)? *Prompt for more detail about level of acceptability (e.g., What is the most you would be willing to pay to access the site?).*
4. What are the benefits you see for other people in using the website?

1. How would you suggest promoting the website to people like yourself (or others)?
2. Do you have any further comments about the website?
