# Supplementary material for: User Views on Online Sexual Health Symptom Checker Tool: Qualitative Research
Source: JMIR Form Res. 2024 Nov 4;8:e54565. doi: 10.2196/54565 (PMC11574491; doi:10.2196/54565)
Supplement: Multimedia Appendix 6 [file formative_v8i1e54565_app6.docx]

**Characteristics of CHECKING MY RISK EASILY AND PRIVATELY from reviewing coded data**

- Starting point for healthcare seeking
- Handy/accessible any time of day/when unable to access a healthcare provider (e.g., rural area)
- Not accessible to everyone/inclusive of gender diverse people and their partners
- Easy/quick/not too many questions/not too many responses
- Not having to pay
- Not having to phone up and ask for advice
- Avoiding being overwhelmed by/having to read a lot of information (e.g., Google)
- Anonymity appealing (i.e., not needing to disclose information to another person) if feeling uncomfortable speaking about it; Demographic questions only
- Mobile app would be more convenient
- Avoiding people seeing in on my phone (as an app)
- Older people are more reluctant to talk about their sexual health

**Dimensions**

- Temporal - starting point, quick and easy, single point of information
- Temporal, geographical and financial accessibility - at any time of day, without travelling, as an app
- Inclusivity - not accesible to gender diverse persons
- Social - avoiding judgement by accessing information online or avoiding others seeing it as an app on phone

**Distinct from**

Being concerned, trusting the result.

**Possible consequences**

- Accessing information and advice more easily
- Accessing information through sources other than HCPs

**Summary:**

The main advantages of the MySTIRisk application described by participants related to the convenience and privacy of accessing information online. The application was similar to internet search engines in being potential starting point for healthcare seeking, accessible at any time of day without the need to travel. Moreover, participants saw the tool as overcoming discomfort some might experience in phoning a sexual health clinic or asking a friend. Whilst several noted a smartphone app may further improve the site's convenience for frequent users, concerns about people the app on their phone screen would influence their comfort in downloading it.

The advantage of the application over "Dr Google" is that it provided a single point of information from which to get more targeted sexual health information.

For most participants the questions asked were "straightforward," and the number of questions and responses limited enough so as to avoid unnecessary user burden. However, gender diverse participants and their partners found responses provided limited their trust in the tool's result or prevented them completly from using the tool.
